# 3D Structure and Function of Glycosyltransferases Involved in *N*-glycan Maturation

**DOI:** 10.3390/ijms21020437

**Published:** 2020-01-09

**Authors:** Masamichi Nagae, Yoshiki Yamaguchi, Naoyuki Taniguchi, Yasuhiko Kizuka

**Affiliations:** 1 Graduate School of Pharmaceutical Sciences, The University of Tokyo, Hongo 7-3-1, Bunkyo-ku, Tokyo 113-0033, Japan; 2 Faculty of Pharmaceutical Sciences, Tohoku Medical and Pharmaceutical University, Miyagi 981-8558, Japan; yyoshiki@tohoku-mpu.ac.jp; 3 Department of Glyco-Oncology and Medical Biochemistry, Osaka International Cancer Institute, 3-1-69 Otemae, Chuo-ku, Osaka 541-8567, Japan; tani52@wd5.so-net.ne.jp; 4 Center for Highly Advanced Integration of Nano and Life Sciences (G-CHAIN), Gifu University, 1-1 Yanagido, Gifu 501-1193, Japan

**Keywords:** *N*-glycosylation, glycosyltransferase, atomic structure, GT-A fold, GT-B fold

## Abstract

Glycosylation is the most ubiquitous post-translational modification in eukaryotes. *N*-glycan is attached to nascent glycoproteins and is processed and matured by various glycosidases and glycosyltransferases during protein transport. Genetic and biochemical studies have demonstrated that alternations of the *N*-glycan structure play crucial roles in various physiological and pathological events including progression of cancer, diabetes, and Alzheimer’s disease. In particular, the formation of *N*-glycan branches regulates the functions of target glycoprotein, which are catalyzed by specific *N*-acetylglucosaminyltransferases (GnTs) such as GnT-III, GnT-IVs, GnT-V, and GnT-IX, and a fucosyltransferase, FUT8s. Although the 3D structures of all enzymes have not been solved to date, recent progress in structural analysis of these glycosyltransferases has provided insights into substrate recognition and catalytic reaction mechanisms. In this review, we discuss the biological significance and structure-function relationships of these enzymes.

## 1. Introduction

**Figure 1:**
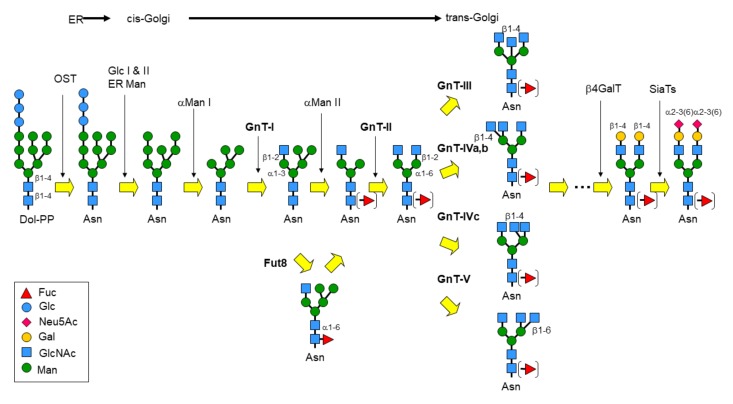
Schematic drawing of *N*-glycan processing. The oligosaccharides comprising 14 sugars were first transferred to an Asn residue, and the *N*-glycans were subsequently processed and matured by the ordered actions of various glycosyltransferases and glycosidases. Monosaccharide symbols follow the symbol nomenclature for glycans (SNFG) system [15].

Protein glycosylation is a ubiquitous post-translational modification found in many organisms. Glycans on proteins have diverse physiological functions and are critically involved in various biological phenomena, including early development, immunity, and neural plasticity [1]. Among the various types of glycosylation, *N*-glycosylation is most ubiquitous in eukaryotes. Over 7000 human proteins are *N*-glycosylated [2], and *N*-glycans with huge structural diversity regulate the various functions of proteins such as folding, trafficking, interactions with other proteins, and enzyme activity [3]. *N*-glycans (Glc_3_Man_9_GlcNAc_2_) are covalently attached onto the asparagine residue of the consensus sequence (N-X-S/T, X≠P) of nascent glycoproteins by oligosaccharyltransferase (OST) complex in the endoplasmic reticulum (ER). Subsequently, the *N*-glycan is extensively modified during protein transport through the secretory pathway by various glycosidases and glycosyltransferases [4]. In the ER, three glucose and one mannose residues are first removed by glucosidase I, II and ER α-mannosidase (Figure 1). Further maturation of *N*-glycans occurs along the secretory pathway, and a properly folded glycoprotein moves through the Golgi apparatus to its final destination. *N*-glycan maturation in the Golgi apparatus is roughly divided into three steps: branch formation, elongation, and capping. All of these steps are catalyzed by the concerted and competitive actions of specific glycosyltransferases and glycosidases, and *N*-glycans with enormous structural diversity are produced, which critically regulate various physiological and pathological events. In this review, we focus on the glycosyltransferases responsible for branch formation of *N*-glycans. In mammalian cells, another major type of glycan, *O*-glycan, is also attached to Ser/Thr residues and regulates various protein functions. This type of glycan is also biosynthesized by the concerted actions of glycosyltransferases in the Golgi and involved in many physiological and pathological phenomena as reviewed by several papers [1,4].

At the initial stage of the *N*-glycan maturation pathway, several mannose residues are trimmed by Golgi α-mannosidase I [5] that generates a key intermediate structure, Man_5_GlcNAc_2_ (Figure 1). The first step in *N*-glycan branching is the addition of a β1-2GlcNAc to the core α1-3 arm mannose residue by *N*-acetylglucosaminyltransferase I (GnT-I, *MGAT1*) [6], which converts oligomannose type glycan to the hybrid-type. GlcNAcMan_5_GlcNAc_2_ is further remodeled to yield the complex-type *N*-glycan by cleavage of the terminal α1-3 linked mannose and α1-6 linked mannose residues by Golgi α-mannosidase II (*MAN2A1* and *MAN2A2*) [7]. Then, it is followed by addition of the second β1-2GlcNAc branch on the core α1-6 linked mannose residue by *N*-acetylglucosaminyltransferase II (GnT-II, *MGAT2*) [8]. Several enzymes further increase branches of *N*-glycans and are the main focus of this review, including *N*-acetylglucosaminyltransferase (GnT)-III (*MGAT3*) [9], GnT-IVa,b (*MGAT4A,B*) [10], GnT-V (*MGAT5*) [11], GnT-VI (*MGAT4C*) [12], GnT-IX (*MGAT5B*) [13], and α1,6-fucosyltransferase (*FUT8*) [14] (Figure 1). After branch formation, complex-type glycans are further extended and capped basically with galactose, sialic acid, and fucose residues, resulting in a high-level of complexity (Figure 1).

The branch formation of *N*-glycans is vital for various protein functions. Deletion of the genes encoding these glycosyltransferases in mice results in postnatal defects of the immune system [16,17] and nervous system [18] as well as various disease-related phenotypes in cancer [19], Alzheimer’s disease [20], diabetes [21], and chronic obstructive pulmonary disease (COPD) [22]. These phenotypes are mediated by specific target glycoproteins that would have carried specific branches. Although enzymatic assays of substrate specificities for oligosaccharides have been extensively studied, it remains unclear how these glycosyltransferases act on target proteins in the Golgi apparatus. Elucidating the mechanisms of protein selectivity of these enzymes would provide more insights into how *N*-glycan branches function in vivo.

In general, glycosyltransferases catalyze synthesis of glycosidic linkages by transfer of a sugar residue from a high energy donor to a specific acceptor molecule. The structural analyses of glycosyltransferases are an effective approach to clarify substrate recognition and catalytic reaction mechanisms. Structural analyses also clarify the selective reaction mechanisms of glycosyltransferases for target glycoproteins, leading to the rational design of glycan-based inhibitors. To date, the 3D structures of glycosyltransferases have been roughly grouped into three folds, designated as GT-A, GT-B, and GT-C [23,24]. In particular, 3D structures of nucleotide sugar-dependent glycosyltransferases have been classified into only two types of folds, GT-A and GT-B, with slight variations. GT-A and GT-B topologies contain one or two Rossmann fold domains. The GT-A fold contains a single Rossmann fold and usually has the Asp-x-Asp (DxD) motif. The side chain carboxylates coordinate a divalent cation such as Mn^2+^ or Mg^2+^. In contrast, the GT-B fold consists of two Rossmann folds of similar size separated by a large cleft. Active site is located in the cleft and stabilized by two long C-terminal α-helices [25]. The catalytic reaction of GT-B fold enzymes is typically metal independent. GT-C fold enzymes are membrane embedded proteins and utilize lipid phosphate sugar donors. The catalytic center of GT-C fold enzymes resides between soluble and transmembrane domains. Glycosyltransferases involved in *N*-glycan maturation have GT-A or GT-B folds.

In this review, we focus on the structure-function relationship of glycosyltransferases involved in *N*-glycan maturation especially for branch formation (Table 1). 3D structural analyses of some of these enzymes have clarified their substrate specificities and catalytic reaction mechanisms. Further structural determination of the remaining enzymes will be important challenges in the future.

## 2. Structure and Function of Glycosyltransferases for *N*-Glycan Branching

### 2.1. Structural and Functional Overview of GnT-I

GnT-I, encoded by the *MGAT1* gene, is an inverting glycosyltransferase. The α-linked GlcNAc moiety from the UDP-α-GlcNAc donor is transferred to the Manα1-3Manβ-R arm (α1-3 branch) of the Man_5_GlcNAc_2_-Asn acceptor, creating the β-linked GlcNAcβ1-2Manα1-3Manβ-R product [31,32,33]. GnT-I belongs to the GT13 family in the CAZy database [34]. The transfer of the initial GlcNAc converts oligomannose glycan to hybrid and complex glycans. Many of the enzymes in the *N*-glycan biosynthetic pathway require the prior action of GnT-I [31,32].

*Mgat1* knockout mice die by E9.5 with defects in growth and morphogenesis [35,36], which clearly indicates that complex glycan structures are essential for development of higher organisms. Conditional knockout of the *Mgat1* gene has also been conducted in mice. Oocyte-specific [37] and spermatogonia-specific [38,39] deletions of *Mgat1* have revealed that GnT-I-producing *N*-glycans are required for proper development of germ cells. Since GnT-I is considered to virtually act on almost all *N*-glycosylated proteins, it is difficult to identify the modified glycoproteins that are mainly involved in a phenotype. Notably, in mammalian testes, an inhibitory protein of GnT-I is specifically expressed, which is designated as GnT-1IP (*MGAT4D*). Expression of GnT-1IP compromises the biosynthesis of hybrid and complex *N*-glycans, which was shown to be involved in germ cell-Sertoli cell interactions [40].

Since *MGAT1* knockout mammalian cells are viable, disruption of the *MGAT1* gene is often used for production of glycoproteins with less complexity of glycans in the bioengineering field [41,42]. Loss of GnT-I makes all *N*-glycans oligomannosidic, which are easily cleaved by endoglycosidase H. This strategy is useful for crystallization of glycoproteins in structural biology [43].

**Figure 2:**
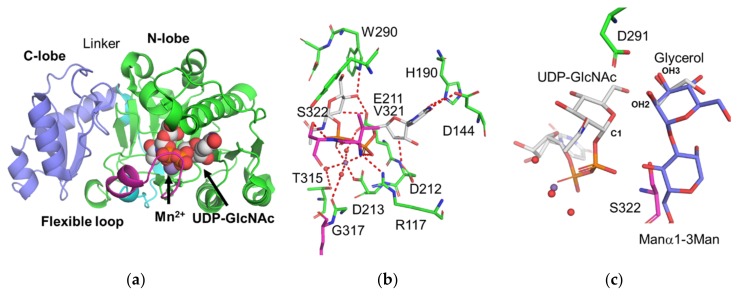
Crystal structure of rabbit GnT-I. (**a**) Crystal structure of rabbit GnT-I in complex with UDP-GlcNAc and a Mn^2+^ ion (PDB code: 1FOA). Protein and ligands are shown as ribbon and sphere models, respectively. The N-lobe (106–317), flexible loop (318–330), linker (331–353), and C-lobe (354–447) are green, magenta, cyan, and blue, respectively. The colors of sphere models are as follows: carbon (grey), oxygen (red), phosphorus (orange) and manganese (purple). (**b**) Close up view of the UDP-GlcNAc binding site. Amino acid residues that interact with UDP-GlcNAc or the Mn^2+^ ion are shown as rod models. Mn^2+^, and three water molecules which coordinate with Mn^2+^ ion are shown in sphere models. The hydrogen and coordination bonds are shown in red dotted lines. (**c**) Putative acceptor-binding mode. The Manα1-3Man unit (colored in blue) is superimposed onto the glycerol molecule observed in GnT-I UDP-CH_2_-GlcNAc complex (PDB code: 2AM3). The side chain of D291 is located between the C1 atom of GlcNAc and 2-OH of mannose. S322 interacts with both the donor and acceptor substrates and thus may be involved in the product release step.

Crystal structures of rabbit GnT-I in the apo form and the presence of Mn^2+^ and UDP-GlcNAc have been determined at 1.5 and 1.8 Å resolutions, respectively [26]. The overall structure of rabbit GnT-I is classified as a GT-A fold (Figure 2a). The GnT-I structure contains an N-terminal core Rossmann fold with C-terminal extension, which forms an antiparallel β-sheet and α-helical segment. The flexible loop (residues 318–330), which is located adjacent to the nucleotide sugar-binding site, is completely missing in the apo form. However, this loop changes to an ordered structure in the donor- metal- bound form. This loop directly contacts with a donor substrate and acts as the lid of cavity. The UDP-GlcNAc-Mn^2+^ complex structure demonstrates that the uracil ring and ribose of UDP-GlcNAc form hydrogen bonds with GnT-I and is further stabilized by hydrophobic interactions. The Mn^2+^ ion coordinates with α-, β-phosphates, three water molecules, and the side chain of D213 in octahedral geometry (Figure 2b). Many GT-A fold enzymes require a divalent cation (usually Mn^2+^) coordinated by a DxD motif to interact with the phosphodiester of the sugar nucleotide donor. In the case of GnT-I, the DXD motif corresponds to E211-D212-D213. Although these three residues are exposed and point toward the donor substrate, D213 is the sole residue to directly coordinate with the metal ion. For an inverting glycosyltransferase, the general base is proposed to assist deprotonation of the nucleophilic hydroxyl of the acceptor. D291 is a strong candidate for the general base because it is the only residue that is 4.7 Å from the GlcNAc C1 atom. Although the acceptor substrate is absent in the donor analog complex, the glycerol molecule resides in the putative acceptor-binding site [27]. The position and orientation of the glycerol provide the hypothetical model of the acceptor disaccharide unit (Manα1-3Man) and useful information of transfer mechanism. In this model, the OH2 and OH3 of mannose at the non-reducing end interact with the side chain of D291 (Figure 2c). The flexible loop can also interact with the acceptor disaccharide and is proposed to play crucial roles in substrate binding and product release.

### 2.2. Structural and Functional Overview of GnT-II

GnT-II encoded by the *MGAT2* gene generates the second GlcNAcβ1-2 branch from the trimannosyl glycan core using UDP-GlcNAc as the sugar donor (Figure 1) [44,45]. In most metazoans, GnT-II is the sole member of GT16 in the CAZy database. Human *MGAT2* deficiency (CDG-IIa) [46] and mice lacking *Mgat2* [47] display similar developmental and postnatal defects. *Mgat2*-knockout mice show early postnatal lethality, and severe locomotor and developmental abnormalities in multiple organs. These results demonstrate that complex type *N*-glycans are essential for mammalian development. Intriguingly, unusual extension of bisecting GlcNAc (described in detail later) with the Lewis^x^ structure was reported to be formed in *Mgat2*-deficient kidneys [47], suggesting that some mechanisms in mammals compensate for loss of the GnT-II-producing branch. Moreover, T cell-specific *Mgat2* knockout upregulates expression of the polylactosamine (polyLacNAc) structure on α1-3 arm to functionally compensate for loss of the LacNAc unit [48]. These findings suggest that mammals have the unique glycan biosynthetic system to adapt to changes in glycan structures.

**Figure 3:**
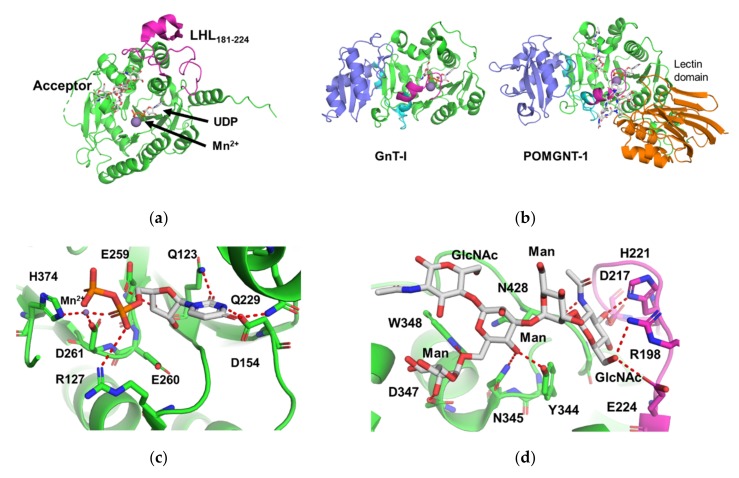

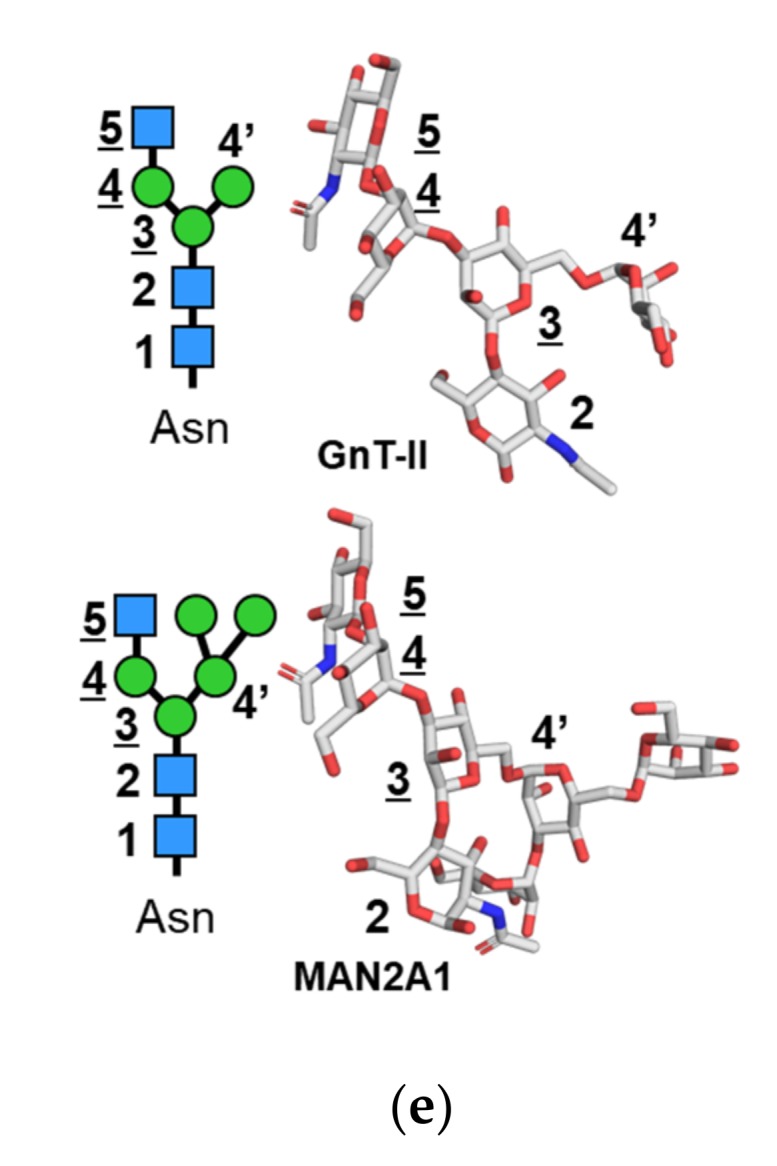
Crystal structure of human GnT-II. (**a**) Putative ternary complex structure of human GnT-II, UDP, and acceptor *N*-glycan. This model was constructed by combining the GnT-II UDP-Mn^2+^ complex (PDB code: 5VCM) and GnT-II acceptor *N*-glycan complex (PDB code: 5VCS). The inserted loop–helix–loop (LHL181-224) is highlighted in magenta. GnT-II, UDP, acceptor glycan and Mn^2+^ are shown in ribbon, stick, stick, and sphere models, respectively. (**b**) Structures of GnT-I (left panel, PDB code: 1FOA) and POMGNT-1 (right panel, PDB code: 5GGI). For comparison, the structures of these two enzymes are depicted at the same viewing angles as Figure 3a. The colors of GnT-I are same with Figure 2a. The lectin domain (97-253), N-lobe (254-502), flexible loop (503-514), linker (515-538) and C-lobe (539-647) of POMGNT-1 are colored in orange, green, magenta, cyan and blue, respectively. POMGNT-1, UDP, acceptor glycopeptide and Mn^2+^ are shown in ribbon, stick, stick and sphere models, respectively. (**c**) Close up view of the UDP binding site (PDB code: 5VCM). The hydrogen and coordination bonds are shown as red dotted lines. The colors of Figure 3c-e are same with Figure 3a. (**d**) Close up view of the acceptor *N*-glycan binding site (PDB code: 5VCS). (**e**) Structural comparison between the acceptor *N*-glycan (GlcNAcMan_3_GlcNAc) bound to human GnT-II (upper panel) and substrate *N*-glycan (GlcNAcMan_5_GlcNAc_2_) bound to fruit fly MAN2A1 (PDB code: 3CZN, lower panel). Schematic drawing of each *N*-glycan is also shown. The sugar residues of “recognition arm” (GlcNAcβ1-2Manα1-3Man, residues 3, 4, and 5) are underlined.

Crystal structures of the human GnT-II catalytic domain UO_2_ derivative, Mn^2+^-UDP complex, and acceptor (GlcNAcMan_3_GlcNAc_2_-Asn) complex were recently determined at 2.0, 1.6, and 2.8 Å resolutions, respectively [28]. The overall fold of human GnT-II consists of an eight-stranded twisted β-sheet with 12 α-helical segments and forms GT-A fold such as GnT-I (Figure 3a). Among many glycosyltransferases with GT-A folds, the overall structure of GnT-II is similar to those of GnT-I and protein *O*-linked mannose β1,2-*N*-acetylglucosaminyltransferase 1 (POMGNT-1) (Figure 3b) [49]. POMGNT-1 transfers GlcNAc to the Man-*O*-Ser/Thr acceptor via the β1-2 linkage to form core M1 (GlcNAcβ1-2Man-*O*-Ser/Thr) or M2 (GlcNAcβ1-2(GlcNAcβ1-6)-Man) glycans. Despite low sequence identities with GnT-II (18% and 17% for rabbit GnT-I and human POMGNT1, respectively) the GT-A fold in all three enzymes is highly conserved (rmsd of 1.5 Å for 737 Cα atoms in GnT-I and 1.3 Å for 793 Cα atoms for POMGNT1 versus GnT-II, respectively) and employs identical or similar amino acids to interact with the UDP portion of the donor substrate. All three enzymes employ an EED sequence for the DxD motif, a conserved Arg residue forming a salt bridge with the α-phosphate of the sugar nucleotide, and a His, Glu, and peptide bond carbonyl that forms hydrogen bonds with the nucleotide and ribose (Figure 3c). GnT-II forms additional hydrogen bonds with the uracil substituents. Although the overall folds of these three enzymes are similar, there are local structural differences. GnT-II has an inserted loop–helix–loop segment (LHL) corresponding to residues 181-224, whereas GnT-I and POMGNT1 have extra C-terminal segments composed of α/β structures.

GnT-II in complex with acceptor pentasaccharide (GlcNAcβ1-2Manα1-3[Manα1-6]Manβ1-4GlcNAc) demonstrates that GnT-II closely interacts with GlcNAc on α1-3 branch, β-mannose, and α1-6 branched mannose residues (Figure 3d). In particular, the GlcNAcβ1-2Man unit of α1-3 branch is inserted into the shallow cavity named the exosite that is distinct from the active site. When GnT-I UDP-GlcNAc complex structure is superimposed onto the GnT-II acceptor complex, the GlcNAc C1 is reasonably positioned for an in-line nucleophilic substitution by the 2-OH hydroxyl of the α1-6-linked mannose acceptor residue. The side chain of D347 forms a hydrogen bond with 2-OH of α1-6-linked mannose and is positioned to act as the catalytic base for deprotonating the nucleophilic hydroxyl, which is consistent with the predicted inverting catalytic mechanism.

The GlcNAcβ1-2Manα1-3Man trisaccharide unit is named as the “recognition arm” (highlighted in Figure 3e) and proposed to be a common binding site for various enzymes such as MAN2A1, GnT-II, GnT-III, GnT-IV, and FUT8 [50]. In fact, the acceptor recognition mode of GnT-II shows striking similarity with the substrate recognition mode of *Drosophila* Golgi α-mannosidase II (MAN2A1) [51], although these two enzymes have different structural folds and catalyze distinct reactions (Figure 3e). In GnT-II and MAN2A1 structures, the exosite interactions with the recognition arm are similar. Moreover, the conformations of the recognition arms themselves are similar in the two structures. 

Crystal structure of the GnT-II acceptor complex well exemplifies the sequential reaction mechanism of *N*-glycan. GnT-II acts on the GlcNAcMan_3_GlcNAc_2_-Asn substrate only after GnT-I and MAN2A1 actions, largely because these latter enzymes complete the recognition arm and unmodified acceptor arm that are critical determinants for interactions with the GnT-II active site. The exosite pocket encloses the terminal GlcNAc residue of the recognition arm, and additional tight interactions with the remainder of the recognition arm explain the inability of the enzyme to act on substrates extended by either B4GALT1 or GnT-III.

### 2.3. Functional Overview of GnT-III

GnT-III encoded by the *MGAT3* gene catalyzes transfer of a GlcNAc residue to β-mannose via the β1-4 linkage to form a so-called “bisecting GlcNAc” structure. GnT-III is classified into GT17 in the CAZy database and was originally purified from the rat kidney [52]. Although various enzymatic and functional studies have been performed regarding bisecting GlcNAc, the crystal structure of GnT-III has not yet been solved.

Bisecting GlcNAc has unique features that differ from those of other GlcNAc branches [53]. First, although bisecting GlcNAc has been reported to be rarely extended in *Mgat2*-deficient mice as described above, it is usually not elongated, whereas other GlcNAc branches in *N*-glycan are further modified with galactose, sialic acid, fucose, and others. The second feature of bisecting GlcNAc is its inhibitory effects on other glycosyltransferases. The enzymes responsible for producing other *N*-glycan branches (e.g., GnT-IV, GnT-V, and FUT8) as well as the enzymes acting on *N*-glycan terminals (fucosyltransferases, sialyltransferases and biosynthetic enzymes for human natural killer-1 epitope) are inhibited partially or completely by the presence of a bisecting GlcNAc in *N*-glycan [54,55,56]. This is probably because the presence of bisecting GlcNAc alters glycan conformation and restricts preferable conformers of *N*-glycan. Molecular dynamic simulation, NMR analysis, and X-ray crystallography suggest that bisected *N*-glycans tend to prefer back-fold conformations in which the α1-6 branch flips back to the reducing end [56,57,58,59,60]. Our MS glycan analysis revealed increases in various terminal epitopes of *N*-glycans in *Mgat3*-knockout brain, which is concomitant with complete loss of bisecting GlcNAc, suggesting that one of the physiological functions of bisecting GlcNAc is to suppress formation of mature and complex *N*-glycans [56]. Similarly, Dr. Gu’s group reported that the level of bisecting GlcNAc is negatively correlated with the level of sialylation in various cell lines [61].

Bisecting GlcNAc is involved in diseases, including cancer and Alzheimer’s disease [62,63]. Overexpression of GnT-III in B16 melanoma cells largely suppresses lung metastasis in mice [64]. This anti-metastatic phenotype is caused by elevated expression of E-cadherin at the cell surface [65]. Moreover, *Mgat3*-knockout mice show rapid tumor growth and metastasis of the MMTV/PyMT breast cancer model [66]. Furthermore, the *MGAT3* expression is down-regulated by induction of epithelial–mesenchymal transition (EMT) that is critical for epithelial cancer metastasis, whereas overexpression of GnT-III suppresses EMT phenotypes [67,68]. These findings suggest that bisecting GlcNAc has anti-tumor functions.

Several reports have shown that GnT-III also promotes cancer growth. *Mgat3*-knockout mice showed reduced cancer growth in chemically induced liver cancer model [69,70]. Furthermore, in ovarian cancer, *MGAT3* was epigenetically upregulated [71,72,73], and the high levels of *MGAT3* are correlated with poor prognosis [74]. Knockdown of *MGAT3* reduced the growth of ovarian cancer in a mouse model, and the modification of Notch1 with bisecting GlcNAc was shown to cause lysosomal degradation of Notch1 and be involved in this cancer-suppressive phenotype [74]. Therefore, bisecting GlcNAc has context-dependent dual roles in cancer malignancy, probably depending on the expression profiles of target glycoproteins and other glycan structures.

Under physiological conditions, *Mgat3* mRNA shows tissue-specific expression with the highest levels in the brain and kidney [75], suggesting that bisecting GlcNAc plays certain roles in these organs. Dr. Endo’s group found upregulation of *MGAT3* mRNA level in Alzheimer’s disease (AD) patient brains [76]. In a mouse AD model, *Mgat3*-knockout results in dramatically reduced deposition of amyloid-β (Aβ) that is the AD-causative aggregation-prone peptide [77]. Consistently, *Mgat3*-deficient AD model mice exhibit better performance in a maze task than wild-type AD model mice. As a mechanism underlying this phenotype, a crucial Aβ-producing enzyme, beta-site APP-cleaving enzyme-1 (BACE1) was revealed to be heavily modified with bisecting GlcNAc in the brain [78], and endosomal localization of BACE1 is altered in *Mgat3*-deficient mice [77]. These findings suggest that bisecting GlcNAc regulates endosomal targeting of target glycoproteins.

GnT-III is proposed to have a GT-A fold, since GnT-III has a DxD motif in its sequence. A structural analysis of GnT-III has not yet been reported. It is notable that GnT-III contains a proline-rich region (residues: 35–86 in human GnT-III) in the juxtamembrane position of GnT-III. This region is located between the transmembrane helix and catalytic domain. The physiological function of this proline rich region is unclear, but it is a unique structural feature of GnT-III. Atomic structural information of GnT-III is strongly desired to reveal the substrate recognition and catalytic reaction mechanisms.

### 2.4. Functional Overview of GnT-IV

GnT-IV catalyzes transfer of a GlcNAc residue to α1-3 linked mannose of the core structure of *N*-glycan via the β1-4 linkage. There are multiple homologous proteins in the GnT-IV family including GnT-IVa, GnT-IVb, GnT-IVc (GnT-VI) and GnT-IVd (GnT-1IP) [79,80,81]. These enzymes belong to GT54 family and are proposed to have GT-A folds. 

GnT-IVa is highly expressed in gastrointestinal tissues, and the involvement of GnT-IVa in type 2 diabetes has been well studied [21,82]. GnT-IVa regulates the functions of *Slc2a2*-encoded glucose transporter 2 (Glut-2) in pancreatic beta cells. Glut-2 is required for both glucose uptake and glucose-stimulated insulin secretion. Impaired functions of Glut2 are induced by high-fat diet administration, which are concurrent with the onset of diabetes [82]. Glycan modification of Glut2 by GnT-IVa enhances the interactions between Glut2 and galectins, leading to prolonged cell surface expression of Glut2 [82]. Furthermore, feeding high fat diet to mice results in transcriptional downregulation of *Mgat4a* [21], which is suggested to be a mechanism for development of diabetes.

GnT-IVb shows the same branching activity as GnT-IVa in vitro with weaker affinity for both donor and acceptor substrates than GnT-IVa [83] and is rather ubiquitously expressed among organs. Double deficient mice of *Mgat4a* and *Mgat4b* have completely abolished GnT-IV activity in all tissues, resulting in the disappearance of the GlcNAcβ1-4 branch on the α1-3 arm [84]. This demonstrates that the only GnT-IVa and -IVb work as active GnT-IV enzymes and that GnT-IVc (GnT-VI) and -IVd do not contribute to the synthesis of the branch.

Human GnT-IVc (*MGAT4C*), also known as GnT-VI and GnT-IV-H, was cloned from the commonly deleted region in pancreatic cancer at 12q21 [85]. This gene is highly expressed in the adult brain. Any enzyme activity of human GnT-IVc has not been detected yet, and the physiological function of this protein is still unclear. A previous report suggested that GnT-IVc might be involved in *N*-glycosylation of CD133 [86], but the detailed actions of GnT-IVc remain to be understood. Interestingly, fish and chicken orthologs of human *MGAT4C* encode GnT-VI enzymes in these species. GnT-VI catalyzes transfer of GlcNAc to the OH4 position of the Manα1-6 arm of the core structure of *N*-glycan, forming the most highly branched pentaantennary glycans. Chick GnT-VI was purified and cloned from the hen oviduct [81,87]. In mammalian tissues, the presence of GnT-VI activity and its product glycans have not been firmly confirmed so far. GnT-IVd (*MGAT4D*; also known as GnT1IP), as described above, binds to GnT-I and inhibits GnT-I activity, thereby blocking the production of complex *N*-glycan [88]. Based on the amino acid sequences, GnT-IVd lacks the C-terminal half of GnT-IVa.

Biochemical and structural studies of GnT-IVs have not yet been fully reported. Further studies will be required to understand the entire functions of this enzyme family, particularly regarding the 3D structures of GnT-IVa and IVb and the biochemical activities of GnT-IVc and -IVd in mammals.

### 2.5. Structural and Functional Overview of GnT-V

GnT-V encoded by the *MGAT5* belongs to the GT18 family in CAZy and catalyzes addition of β1-6 linked GlcNAc to α1-6 linked Man of the *N*-glycan core to form tri- or tetra-antennary branches [89]. GnT-V was initially purified from the rat kidney [90] and a human lung cancer cell line [55], and is now well known as a cancer-related glycosyltransferase. As described below, *Mgat5*-knockout mice show various phenotypes in their immune and nervous systems, indicating that modification of glycoproteins with the β1-6 GlcNAc branch has a wide variety of physiological and pathological functions. GnT-V lacks a conventional Asp-x-Asp (DXD) motif, which is a critical sugar binding motif commonly found in many vertebrate glycosyltransferases. Its catalytic reaction is metal-independent, with weak donor binding and tight acceptor binding [90].

Expression of the *MGAT5* gene in various human cancer types is aberrantly driven by the oncogenic Ras-Raf-ETS pathway [91,92]. Its product glycan, the β1-6 GlcNAc branch, is also upregulated in various cancers, and its levels correlate well with cancer malignancy and a poor prognosis [53,93,94]. Conversely, *Mgat5*-deficient mice show reduced cancer growth in a mammary tumor mouse model [19]. Mechanistically, functional modifications of adhesion molecules (integrins and cadherins), matriptase, tissue inhibitor of metalloproteinase-1 (TIMP-1), and growth factor receptors by GnT-V have been reported to be involved in cancer malignancy [63,95]. For example, formation of the β1-6 GlcNAc branch on adhesion molecules lowers their adhesive properties, leading to enhanced cell migration and metastasis. Furthermore, GnT-V-mediated glycosylation of growth factor receptors promotes their associations with galectins, leading to prolonged residency at the cell surface and augmentation of their downstream signaling [96,97]. These findings indicate that GnT-V is a reasonable drug target for cancer treatment, and our recent findings of GnT-V crystal structure will facilitate structure-based design of GnT-V inhibitors. 

In terms of immunity, glycosylation of the T cell receptor (TCR) by GnT-V has been reported to negatively regulate its function, and T cells from *Mgat5*-knockout mice show enhanced TCR signaling [16]. This was found to be mediated by enhanced galectin binding to TCR glycans. Consistently, *Mgat5*-deficient mice are more susceptible to experimental autoimmune encephalomyelitis than wild-type mice. In terms of the nervous system, *Mgat5*-deficient mice exhibit reduced depression-like phenotypes [98,99], suggesting that the GnT-V-producing branch has physiological functions in the brain. Although the molecular mechanisms and target glycoproteins of these brain phenotypes have not been elucidated, aberrant spine morphology observed in *Mgat5*-deficient neurons may be related [99].

Notably, the activity of GnT-V in cells is regulated by proteolytic cleavage. Signal peptide peptidase-like 3 (SPPL3) has been unambiguously identified as the responsible protease [100]. GnT-V is cleaved by SPPL3 around the C-terminal end of the transmembrane domain, and the cleaved GnT-V is shed into the extracellular space, resulting in reduced cellular activity of GnT-V and its product glycans. Because soluble GnT-V has a non-enzymatic angiogenic activity [101], regulation of GnT-V cleavage by SPPL3 affects cellular glycosylation and cancer biology. 

**Figure 4:**
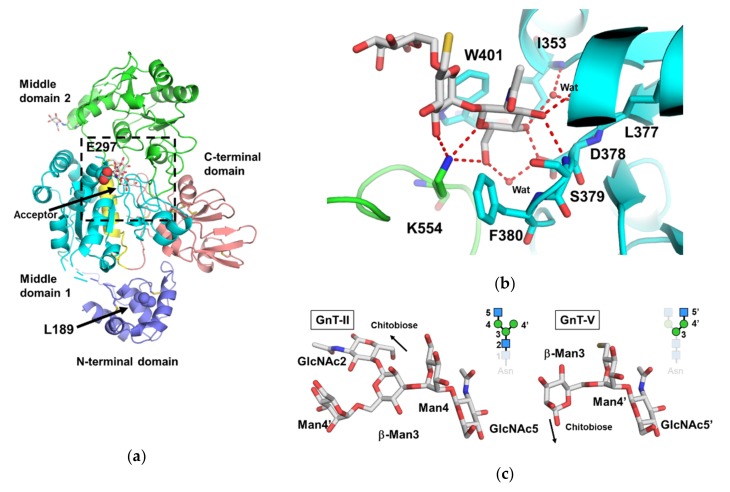
Crystal structure of human GnT-V. (**a**) Overall structure of the human GnT-V luminal domain (PDB code: 5ZIB). The N-terminal domain, middle domain 1, 2, and C-terminal domain are colored in blue, cyan, green, and pink, respectively. The short insertion in middle domain 1 is colored in yellow. The position of the acceptor *N*-glycan was obtained by superposition of the mini-GnT-V-glycan complex (PDB code: 5ZIC). Two important residues (L189 and E289) denoted in the text are shown as sphere models. The oxygen atoms of E289 are highlighted in red. The putative catalytic center is highlighted in the black dotted box. (**b**) Close up view of the acceptor *N*-glycan binding site (PDB code: 5ZIC). The direct and water-mediated hydrogen bonds are shown in red dotted lines. Two aromatic residues (F380 and W401), which may determine the branch specificity of GnT-V, are also shown. (**c**) Structural comparison between the GnT-II acceptor complex and GnT-V acceptor complex. The superposition is based on GlcNAc residues of both branches (GnT-II: α1-3 branch, GnT-V: α1-6 branch). The disaccharide units (GlcNAcβ1-2Man) of the two structures are well superimposable.

Human GT-V is a type II membrane protein consisting of 741 amino acids. The luminal domain of GnT-V was originally predicted as H31-L741. Crystal structures of human GnT-V luminal domain (T121-L741) were recently determined at 1.9 Å resolution (Figure 4a) [29]. The overall structure of GnT-V has a GT-B fold with two accessory domains and N- and C-terminal domains. The structure of GnT-V is in marked contrast to other mammalian GlcNAc transferases such as GnT-I, GnT-II, POMGNT1, and C2GnT-L, which are classified as GT-A folds. Instead, the overall fold of GnT-V is similar to those of bacterial glycosyltransferases in the GT4 family such as mannosyltransferase Wbaz-1 (PDB code: 2F9F), BaBshA (PDB code: 2JJM) and phosphatidylinositol mannosyltransferase (PDB code: 2GEJ). Interestingly, these bacterial glycosyltransferases are retaining enzymes, although GnT-V is an inverting enzyme. In GT-B folds, the catalytic center often resides in a large cleft between two Rossmann fold domains [102]. Thus, point mutations were introduced in the acidic residues around this region. E297 is the strongest candidate for the catalytic residue deduced from the mutational analysis (red dotted box in Figure 4a). 

Regarding the accessary domain of GnT-V, the unique function of the N-terminal helical domain of GnT-V should be noted. This domain is independent of the catalytic reaction, because deletion of this domain still retains full enzymatic activity. Rather, this domain plays an important role in the subcellular localization of GnT-V. A CHO-derived cell line with the L188R mutation in GnT-V is known as the Lec4A mutant, which decreases surface expression of β1-6 branched *N*-glycan by changing the intracellular localization of GnT-V from Golgi to ER [103,104]. The corresponding L189 in human GnT-V is located in the hydrophobic core of the N-terminal domain. Thus, replacement of L to R may impair the local folding of this domain (Figure 4a). The mechanism of GnT-V localization remains unclear, but deep functional analyses of this domain could lead to clarification of the novel transport machinery of glycosyltransferases.

To obtain a ligand complex structure, a trimmed construct named mini-GnT-V was designed and crystallized with a bisubstrate type inhibitor that includes both donor and acceptor analogues connected by a short linker [105]. The structure of the mini-GnT-V inhibitor complex was determined at 2.1 Å resolution. In the complex structure, the electron density of the donor moiety is missing, but the electron density of the acceptor moiety corresponding to the trisaccharide unit, GlcNAcβ1-2Manα1-6Man, is clearly visible (Figure 4b). The GlcNAc residue at the non-reducing end is deeply buried inside the catalytic cavity, while mannose at the reducing end is exposed to solvent. Interestingly, two aromatic rings, F380 and W401, sandwich the GlcNAc residue and the trisaccharide unit rides on the tryptophan side chain. GnT-V transfers GlcNAc only to α1-6 branched mannose, not α1-3 branched mannose. W401 appears to define the branch specificity, because the α1-3 branch, GlcNAcβ1-2Manα1-3Man sterically clashes with the β-mannose and W401.

GnT-II interacts with the “recognition arm” (GlcNAcβ1-2Manα1-3Man) of the α1-3 branch, whereas GnT-V binds to another recognition arm (GlcNAcβ1-2Manα1-6Man) of the α1-6 branch. In both cases, terminal GlcNAc residues are buried inside the cavities, indicating that the extension of galactose inhibits GlcNAc transfer. Interestingly, the conformations of the GlcNAcβ1-2Man unit are similar to each other, suggesting that the two enzymes recognize one of the stable conformations of the disaccharide unit (Figure 4c). The formation of bisecting GlcNAc catalyzed by GnT-III completely inhibits further β1-6 branching by GnT-V [106]. When a bisecting GlcNAc residue is added on β-mannose, this residue lies close to OH6 of the α1-6 branched mannose (Man-2) and sterically clashes with Man-2. In addition, bisected glycan prefers to take extend-b and back-fold conformations, rather than the extend-a conformation that is observed in the complex structure [56,57,58,59,60]. Structural superposition of these two conformations shows that they cannot fit into the surface groove of GnT-V without severe steric clashes. These results explain why the introduction of bisecting GlcNAc prevents β1-6 branch formation.

The β1-6 branch formation catalyzed by GnT-V regulates the physiological activities of substrate glycoproteins such as TCR, integrins, growth factor receptors, and cadherins [16,107]. The β1-6 branch formation of some substrates such as E-cadherin and CEACAM6 is known to occur in a glycosylation site-specific manner [108,109]. A docking model of GnT-V E-cadherin complex suggests that the narrow catalytic cavity of GnT-V is a strong determinant for modification.

### 2.6. Functional Overview of GnT-IX (GnT-Vb)

GnT-IX (GnT-Vb) encoded by the *MGAT5B* gene is a sister enzyme of GnT-V and shares 42% amino acid sequence identities in humans [110]. The GnT-IX gene is exclusively expressed in the brain through epigenetic mechanisms [111]. GnT-IX was originally cloned as a homologous enzyme to GnT-V [110,112], and this enzyme was first found to have unusual *N*-glycan branching activity to form GlcNAcβ1-6Manα1-3Man. However, the presence of this *N*-glycan branch in mammals has not yet been surely confirmed. Rather, subsequent studies using knockout mice of the *Mgat5b* gene have revealed that GnT-IX transfers GlcNAc residue to the 6-position of core Man in *O*-mannose glycan and generates the core M2 (GlcNAcβ1-2[GlcNAcβ1-6]Man) glycan [113].

Although α-dystroglycan (α-DG) is well known to be modified with *O*-mannose glycans, it is a minor among *O*-mannosylated proteins in the mammalian brain [114]. Instead, other glycoproteins, including PTPRZ (or its secreted form called phosphacan), CD24, mainly carry *O*-mannose glycans, and glycomic analyses have shown that their *O*-mannose glycans have a β1,6-GlcNAc branch generated by GnT-IX [115,116,117]. *Mgat5b*-deficient mice lack most of branched *O*-mannose glycans in their brain [118], and additional knockout of *Mgat5* leads to complete loss of this branch, indicating that β1,6-GlcNAc branching of *O*-mannose glycans is mediated dominantly by GnT-IX and only marginally by GnT-V. Functionally, GnT-IX-producing glycans are involved in recovery of myelin damage [119]. Myelination by oligodendrocytes is essential for rapid neuronal transmission, and damage to myelin (demyelination) causes various types of demyelinating disorders, including multiple sclerosis [120]. *Mgat5b*-knockout mice exhibit fast recovery after chemically induced demyelination, compared with wild-type mice [119]. This is probably caused by reduced activation of astrocytes in the corpus callosum of the knockout mice. Involvement of PTPRZ in this phenotype has been suggested, but the detailed mechanisms remain to be elucidated in terms of how β1,6-GlcNAc branched *O*-mannose glycans regulate recovery from demyelination. Upregulation of *MGAT5B* in prostate cancer has also been reported [121]. However, the mechanisms and biological significance of this upregulation have not yet been clarified.

Because of the high sequence identities, the overall structure of GnT-IX may be similar to that of GnT-V. However, a local difference must be present. For example, the enzymatic activity of GnT-IX is enhanced by addition of Mn^2+^ ion [122]. Thus, the donor recognition mechanism of GnT-IX is supposed to be different from that of GnT-V. Further structural analysis of GnT-IX will clarify the metal dependent catalytic mechanism.

### 2.7. Structural and Functional Overview of FUT8

FUT8 is an α1-6 fucosyltransferase that was originally isolated from porcine brain [123] and human gastric cancer cells [124]. FUT8 transfers a fucose residue from a donor substrate, GDP-β-L-fucose, to the innermost GlcNAc of *N*-glycans. The attached fucose is designated as the core fucose, and this structure is commonly observed in many *N*-glycans and profoundly involved in various physiological and pathological processes.

*Fut8* knockout mice show semi-lethality in the C57BL/6 background [125], impaired synaptic plasticity [18], and spontaneous development of emphysema-like lung dysfunction through disturbance of TGF-β and EGF signaling [126]. Furthermore, *Fut8* null mice show abnormalities in their immune system, such as impaired antigen recognition and signaling of the B cell receptor [127], defects in T cell receptor signaling [128], and impaired CD14-dependent Toll-like receptor 4 signaling in macrophages [129,130]. These findings demonstrate that core fucose regulates a wide variety of cellular functions.

In cancer, aberrantly elevated expression of *FUT8* is correlated with poor clinical outcomes of non-small-cell lung cancer patients [131,132]. More recently, a systems biology approach also revealed that FUT8 is a driver of melanoma metastasis [133]. These reports suggest that FUT8 is a therapeutic target for these cancers. In terms of cancer treatment, removal of core fucose from therapeutic IgG drastically enhances (~100-fold) antibody-dependent cellular cytotoxicity (ADCC) [134,135], and this technique is now used clinically. Furthermore, core fucose modification of programmed cell death-1 (PD-1) has been found to be essential for its surface expression on T cells, which also demonstrates that FUT8 is involved in anti-tumor immunity [136]. These findings highlight the clinical importance of the core fucose modification in mammals. 

**Figure 5:**
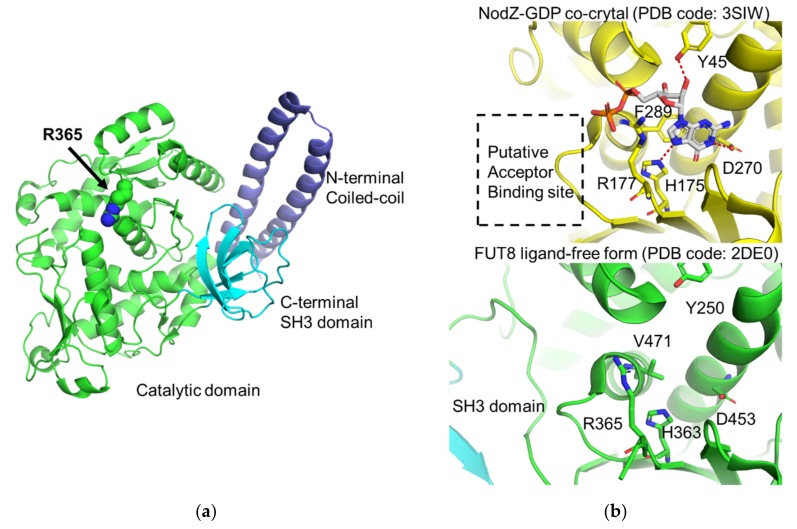
Crystal structure of the human FUT8. (**a**) Overall structure of human FUT8 catalytic domain (PDB code: 2DE0). N-terminal, catalytic and C-terminal SH3 domains are colored with blue, green, and cyan, respectively. The side chain of R365, which is the putative catalytic center is shown in sphere model. The nitrogen atoms of R365 are highlighted in blue. (**b**) Structural comparison between bacterial NodZ-GDP complex (upper panel, PDB code: 3SIW) and human FUT8 in ligand-free form (lower panel). The putative acceptor-binding site is depicted as a black-dotted box. The GDP and amino acid residues which interact with GDP in NodZ are shown in stick models. The hydrogen bonds are depicted in red dotted lines. The carbon atoms of NodZ and GDP, nitrogen, oxygen and phosphorus atoms are colored in yellow, grey, blue, red, and orange, respectively. The corresponding residues Fut8 are also shown.

Crystal structure of the human FUT8 catalytic domain in its unliganded form was determined at 2.6 Å resolution [30]. The overall structure of human FUT8 forms a GT-B like fold with an N-terminal coiled-coil domain and C-terminal SH3 domain (Figure 5a). The catalytic core of FUT8 shows structural similarity with the canonical GT-B fold, but the large part of the N-terminal Rossmann fold is lacking. A DALI search indicates that the 3D structure of FUT8 shows close similarity with those of plant α1-2 fucosyltransferase *At*FUT1 (Z-score: 17.7–18.2) [137,138], *Bradyrhizobium* sp WZ9 α1-6 fucosyltransferase NodZ (Z-score: 15.4-15.7) [139,140], *Caenorhabditis elegans* POFUT2 (Z-score: 15.6) [141], human POFUT2 (Z-score: 14.6-15.1) [142], mouse POFUT1 (Z-score: 13.8-14.1) [143], and human POFUT1 (Z-score: 13.9-14.0) [144]. Although these enzymes are classified into various CAZy families: FUT8 and NodZ (GT23), AtFUT1 (GT37), POFUT1 (GT65), and POFUT2 (GT68), the overall structures are quite similar. These fucosyltransferases commonly use GDP-Fucose as donor substrates. Thus, the donor recognition mechanisms are well conserved. Structural comparison with rhizobial NodZ belonging to the same GT23 family has provided the putative donor recognition mechanism of FUT8 [140]. The amino acid residues that interact with the GDP moiety are shared between these two enzymes (Figure 5b).

Compared with common donor recognition mechanisms, the acceptor substrate recognition of fucosyltransferases exhibits a wide variety of interaction modes. For example, *At*FUT1 puts α1-2 linked fucose onto galactose in xyloglucan oligosaccharide, whereas POFUT1 and 2 transfer *O*-linked fucose to the epidermal growth factor-like (EGF) repeat and thrombospondin type 1 (TSR) repeat, respectively. Structural superposition of these enzymes suggests that the acceptor binding site of FUT8 is supposed to be located at the large cleft around the SH3 domain.

Based on the docking model of FUT8 in complex with donor and acceptor substrates, FUT8 is proposed to employ a single-step S_N_2 mechanism with base-catalyzed deprotonation of the acceptor nucleophile [145]. In the proposed cycle, R365 plays crucial roles in the binding and stabilization of donor GDP-fucose (Figure 5b).

For the two accessary domains of FUT8, the physiological functions remain unclear. The N-terminal coiled-coil domain is located at the opposite side of the catalytic center, indicating that the physiological function of this region may be independent of enzymatic activity. The C-terminal SH3 domain is a unique structural feature of human FUT8, although the physiological function of the FUT8 SH3 domain is also unknown. Because it lacks the several critical residues that interact with the proline-rich peptide [146], it may have another function.

## 3. Conclusions

In this review, we overview the functional and structural aspects of mammalian glycosyltransferases involved in *N*-glycan maturation. For the past two decades, the functional importance of these enzymes was elucidated using overexpression/knockout cells and animals. Furthermore, alterations of the enzyme levels and their glycan products in various human disorders have been found. In contrast to such progress in functional studies, structural information of these enzymes is still limited. In particular, the atomic details of acceptor substrate recognition are largely unknown yet. It is unclear how these enzymes selectively act on their target proteins. In living cells, many other factors may be involved in regulation of their activity, such as sub-Golgi localization and complex formation. Without fine structural information, we cannot fully elucidate how these enzymes recognize their substrates to produce glycans. Determination of atomic structures of the enzymes would lead to new research directions, such as structure-based design of glycosyltransferase inhibitors or creation of new mutants having neofunctions. We believe that structural glycobiology is an essential part of glycoscience for understanding the expression and function of glycans in humans.

## Figures and Tables

**Table 1:** Summary of 3D structures of glycosyltransferases involved in *N*-glycan maturation. All these data are deposited in Protein Data Bank.

Enzyme	CAZy Family	Fold	Mechanism	Ligand	PDB Code	Reference
Rabbit GnT-I	GT13	GT-A	Inverting	UDP-GlcNAc, Mn^2+^	1FOA	[26]
				(CH_3_-Hg derivative)	1FO8	
				-	1FO9	
Rabbit GnT-I				UDP-CH_2_-GlcNAc, Mn^2+^	2AM3	[27]
				^1^ UDP-2F-Glc, Mn^2+^	2AM4	
				UDP, Mn^2+^	2AM5	
				UDP-GlcNAc phosphonate, Mn^2+^	2APC	
Human GnT-II	GT16	GT-A	Inverting	UDP, Mn^2+^	5VCM	[28]
				(UO_2_ derivative)	5VCR	
				Acceptor (GlcNAcMan_3_GlcNAc)	5VCS	
Human GnT-V	GT18	GT-B	Inverting	-	5ZIB	[29]
Human GnT-V (Mini-GnT-V)				Acceptor (GlcNAcMan_2_)	5ZIC	
Human FUT8	GT23	GT-B	Inverting	-	2DE0	[30]

“-”; Ligand free form, ^1^ UDP-2F-Glc; UDP-2-deoxy-2-fluoro-glucose.

## Abbreviations

GlcNAc	*N*-acetylglucosamine
Man	Mannose
GnT	*N*-acetylglucosaminyltransferase
GT	Glycosyltransferase
FUT	Fucosyltransferase
ER	Endoplasmic reticulum
TCR	T-cell receptor
